# Gene expression profiles in COVID-19-associated tracheal stenosis indicate persistent anti-viral response and dysregulated retinol metabolism

**DOI:** 10.1186/s13104-024-06775-y

**Published:** 2024-05-16

**Authors:** Russell Seth Martins, Joanna Weber, Kostantinos Poulikidis, Al Haitham Al Shetawi, M. Jawad Latif, Syed Shahzad Razi, Robert S. Lebovics, Faiz Y. Bhora

**Affiliations:** 1 Department of Surgery, Hackensack Meridian School of Medicine, Hackensack Meridian Health (HMH) Network, 08820 Edison, NJ USA; 2 Division of Surgical Oncology, Department of Surgery, Dyson Center for Cancer Care, Vassar Brothers Medical Center, Nuvance Health, 12601 Poughkeepsie, NY USA; 3 Division of Oral and Maxillofacial Surgery, Department of Surgery, Vassar Brothers Medical Center, Nuvance Health, 12601 Poughkeepsie, NY USA; 4 Division of Thoracic Surgery, Department of Surgery, Hackensack Meridian School of Medicine, Hackensack Meridian Health (HMH) Network– Central Region, 65 James Street, 08820 Edison, NJ USA; 5 Chief of Thoracic Surgery, Hackensack Meridian Health (HMH) Network– Central Region, Hackensack Meridian School of Medicine, 65 James Street, 08820 Edison, NJ USA

**Keywords:** Coronavirus disease 2019, Subglottic stenosis, Molecular medicine, RNA, Endotracheal intubation, Granulation tissue

## Abstract

**Introduction:**

Coronavirus disease 2019 (COVID-19)-associated tracheal stenosis (COATS) may occur as a result of prolonged intubation during COVID-19 infection. We aimed to investigate patterns of gene expression in the tracheal granulation tissue of patients with COATS, leverage gene expression data to identify dysregulated cellular pathways and processes, and discuss potential therapeutic options based on the identified gene expression profiles.

**Methods:**

Adult patients (age ≥ 18 years) presenting to clinics for management of severe, recalcitrant COATS were included in this study. RNA sequencing and differential gene expression analysis was performed with transcriptomic data for normal tracheal tissue being used as a control. The top ten most highly upregulated and downregulated genes were identified. For each of these pathologically dysregulated genes, we identified key cellular pathways and processes they are involved in using Gene Ontology (GO) and KEGG (Kyoto Encyclopedia of Genes and Genomes) applied via Database for Annotation, Visualization, and Integrated Discovery (DAVID).

**Results:**

Two women, aged 36 years and 37 years, were included. The profile of dysregulated genes indicated a cellular response consistent with viral infection (CXCL11, PI15, CCL8, DEFB103A, IFI6, ACOD1, and DEFB4A) and hyperproliferation/hypergranulation (MMP3, CASP14 and HAS1), while downregulated pathways included retinol metabolism (ALDH1A2, RBP1, RBP4, CRABP1 and CRABP2).

**Conclusion:**

Gene expression changes consistent with persistent viral infection and dysregulated retinol metabolism may promote tracheal hypergranulation and hyperproliferation leading to COATS. Given the presence of existing literature highlighting retinoic acid’s ability to favorably regulate these genes, improve cell-cell adhesion, and decrease overall disease severity in COVID-19, future studies must evaluate its utility for adjunctive management of COATS in animal models and clinical settings.

**Supplementary Information:**

The online version contains supplementary material available at 10.1186/s13104-024-06775-y.

## Background

Acquired tracheal stenosis may occur in up to 20% of patients after prolonged intubation [1–3], inflicting significant limitations on patients’ respiratory function, vocal ability, and overall quality of life [4]. During the coronavirus disease 2019 (COVID-19) pandemic, studies from across the world reported intubation rates ranging from 5 to 88% amongst patients with COVID-19 [5]. Moreover, the median duration of intubation may be as long as 17 days, with more than 18% of patients requiring reintubation within one week of extubation [6, 7]. It is believed that in addition to airway mucosal damage due to intubation, tracheitis due to COVID-19 may also contribute to tracheal stenosis [8]. As a result, the European Laryngological Society urges physicians to maintain a high index of suspicion for tracheal stenosis amongst patients with COVID-19 requiring intubation [9].

The management of tracheal stenosis is mainly interventional, with options including tracheal resection and reconstruction, bronchoscopic dilation, laser therapy, spray cryotherapy, or airway stent placement [10, 11]. Adjunctive medical therapies have been explored with limited success, including local mitomycin C, local or inhaled steroids, oral proton pump inhibitors, trimethoprim-sulfamethoxazole, penicillin, and macrolide antibiotics [12–14]. Recently, an increased understanding of molecular and genetic profiles of tracheal granulation tissue is guiding the exploration of novel therapies for tracheal stenosis [15]. However, data is sparse and there is an urgent need to explore genetic mechanisms underlying the development of tracheal stenosis [16], particularly in the context of COVID-19 infection. Thus, we aimed to investigate patterns of gene expression in the tracheal granulation tissue of patients with COVID-19-associated tracheal stenosis (COATS), and leverage gene expression data to identify key dysregulated pathways and processes. We also discuss potential therapeutic options based on the identified gene expression profiles. Although this Research Note presents data for a small sample, we believe the novelty of the results warrant its sharing with the scientific community.

## Main text

### Methods

This study was conducted between July 2020-July 2021 at Nuvance Health in Connecticut, USA, after receiving ethical approval from the institutional review board (ID: 2019-19).

#### Patient enrollment and sample collection

We sought to include adult patients (age ≥ 18 years) presenting to clinics for management of severe, recalcitrant COATS, as visualized by upper airway endoscopy. Patients were diagnosed with COATS if they developed tracheal stenosis in the setting of active COVID-19 infection during airway interventions performed for the management of COVID-19. Informed consent was acquired prior to patient enrolment and data collection. Patient data collected included demographics, baseline health status, and clinical history. Samples were collected at the index visit and subsequent visits at time of reintervention (with each reintervention and sample collection being roughly six months apart), making for a total of five tissue samples (two from patient 1 and three from patient 2).

Tracheal tissue biopsies of granulation tissue were collected at the time of endoscopic intervention, which consisted of balloon dilation and spray cryotherapy. If enrolled patients returned to clinics requiring reintervention for re-stenosis, they were reapproached for collection of additional samples. Additional biopsies were collected from consenting patients, with these being considered as unique samples for analysis.

#### RNA sequencing of samples (transcriptomics)

Tissue samples were sent to Azenta Life Sciences (Burlington, Massachusetts, USA) for processing and RNA sequencing. A next-generation sequencing platform (HiSeq ®; Illumina, Inc., San Diego, California, USA) with Poly(A) selection was used to prepare the cDNA (complementary DNA) libraries.

#### Normal control

Publicly accessible transcriptomics data for normal tracheal tissue was sourced from the public data repositories of the National Center for Biotechnology Information (NCBI: SRR16760102) and the European Nucleotide Archive (ENA: ERR2022844).

#### Data analysis

CLC Genomics Workbench by QIAGEN (Venlo, Netherlands) was used for analysis of the sequenced data. Trimmed reads were aligned and annotated with Ensembl 91: Dec 2017 (GRCh38.p10). Differential gene expressions were explored between tissue samples of COATS and normal control data. Gene expression was considered significant if the false discovery rate (FDR) p-value was < 0.05 and the fold change (ratio of value in specimen to value in normal control) was > 1.5. The top 10 most highly upregulated and downregulated genes were identified by calculating a change coefficient that accounted for both FDR p-value and fold change, as follows:

$$ \left|change\,coefficient\right|= {-log}_{2} FDR p-value \times {log}_{2} fold\,change$$

The protein class of the gene products and their relevant functions were retrieved from the Human Protein Atlas [17], an open-source repository containing data on all proteins coded by the human genome. Based on their relevant functions, we determined whether the upregulation or downregulation of each gene was likely part of the body’s protective response to COVID-19-infection (e.g., immune system activation) or contributing towards pathological mechanisms causing tracheal stenosis. In addition to comparing the COATS samples to normal control data, we also compared the COATS samples against each other.

Gene Ontology (GO) and KEGG (Kyoto Encyclopedia of Genes and Genomes) functional enrichment were applied via DAVID (Database for Annotation, Visualization, and Integrated Discovery) for significantly upregulated and downregulated genes separately. Significantly upregulated and downregulated cellular pathways and biological processes (i.e., those with a Benjamini p-value < 0.05), along with their GO identification numbers, were noted.

## Results

We included two patients in this study. Patient 1 was a 37-year-old woman with obstructive sleep apnea and hypertension who had developed tracheal stenosis two months after undergoing tracheostomy tube placement during COVID-19 infection. She had undergone three prior spray cryotherapy and balloon dilation procedures for tracheal stenosis prior to presenting to our institution for her index visit with our team. She had been diagnosed with obstructive sleep apnea three years ago and hypertension recently. Patient 2 was a 36-year-old woman with interstitial lung disease and a history of smoking who developed tracheal stenosis a month after prolonged intubation during COVID-19 infection. She was diagnosed with interstitial lung disease a year ago. Patient 2 had not undergone any prior procedures for tracheal stenosis. These details are shown in Supplementary File 1 - Table 1.

**Table 1:** Most highly upregulated genes in COVID-19-associated tracheal stenosis *ACOD1*: Aconitate decarboxylase 1; *CASP14*: Caspase 14; *CCL8*: C-C motif chemokine ligand 8; *CXCL11*: C-X-C motif chemokine ligand 11; *DEFB103A*: Defensin beta 103 A; *DEFB4A*: Defensin beta 4 A; *HAS1*: Hyaluronan synthase 1; *IFI6*: Interferon alpha inducible protein 6; *MMP3*: Matrix metallopeptidase 3; *PI15*: Peptidase inhibitor 15.* Gene identified as pathologically upregulated and contributing towards COATS, and a potential therapeutic target

Gene Name	Chromosome	Product	Relevant Functions	Fold Change	Change Coefficient
CXCL11	4	-	• Chemotactic for activated T-cells	49	43.0
PI15	8	Enzyme inhibitor	• Inhibits chronic inflammation and remodeling	26	36.0
CCL8	17	-	• Chemotactic for monocytes and lymphocytes	64	35.5
DEFB103A	8	Transporter	• Antimicrobial	41	29.7
IFI6	1		• Antiviral	17	26.8
MMP3 *	11	Enzyme	• Breakdown of extracellular matrix in tissue remodeling	25	26.3
ACOD1	13	Enzyme	• Antimicrobial and antiviral (suppresses viral genome replication)	58	25.4
CASP14 *	19	Enzyme	• Epidermal differentiation and cornification	29	24.5
HAS1 *	19	Enzyme	• Synthesis of extracellular matrix	29	24.2
DEFB4A	8	Transporter	• Antimicrobial activity	20	24.1
			• Chemotactic for memory T-cells		

The top 10 most highly upregulated and downregulated genes amongst these five tissue samples are shown in Tables 1 and 2, respectively. Significantly upregulated cellular pathways included those related to COVID-19 and other viral infections (influenza A, human papillomavirus, hepatitis C virus, Epstein-Barr virus, and measles virus), cytokine-cytokine receptor interaction, viral protein interaction with cytokine and cytokine receptor, and NOD (nucleotide oligomerization domain)-like receptor signaling pathways (Supplementary File 1 - Table 2). Significantly upregulated biological processes included antiviral defense, innate immunity, inflammatory response, chemotaxis, angiogenesis, and collagen degradation; significantly downregulated biological processes included cell adhesion & ion transport (Supplementary File 1 - Table 3).

**Table 2:** Most highly downregulated genes in COVID-19-associated tracheal stenosis *CAPN14*: Calpain 14; *CRNN*: Cornulin; *CYP2C19*: Cytochrome P450 family 2 subfamily C member 19; *KRT24*: Keratin 24; *RPTN*: Repetin; *SPINK7*: Serine peptidase inhibitor Kazal type 7; *SPRR3*: Small proline rich protein 3; *SLURP1*: Secreted LY6/PLAUR domain containing 1; *TGM3*: Transglutaminase 3; *TMPRSS11B*: Transmembrane serine protease 11B. * Gene identified as pathologically downregulated and contributing towards COATS, and a potential therapeutic target

Gene Name	Chromosome	Product	Relevant Functions	Fold Decrease	Change Coefficient
KRT24	17	Cytoskeletal protein & plasma protein	• Epithelial cell ultrastructure	253	103.4
CYP2C19	10	Plasma protein	• Metabolism of endogenous and exogenous substance	77	91.6
SPRR3 *	1	-	• Keratinocyte differentiation • Retinol metabolism	161	89.0
TMPRSS11B	4	Enzyme	-	194	83.1
RPTN *	1	-	• Involved in keratinocyte cornification	167	71.9
CRNN *	1	-	• Regulates cell proliferation during inflammatory esponse	225	59.7
SPINK7	5	Plasma protein	-	226	58.2
TGM3	20	Enzyme	• Keratinocyte cornification	156	53.1
CAPN14 *	2	Enzyme	• Regulates cell division	54	52.3
SLURP1 *	8	Plasma protein and Transporter	• Downregulates keratin production [37] • Anti-inflammatory [38]	77	49.6

**Table 3:** Potential therapeutic targets for COVID-19-associated tracheal stenosis *CAPN14*: Calpain 14; *CASP14*: Caspase 14; *COX-2*: Cyclooxygenase 2; *CRNN*: Cornulin; *HAS1*: Hyaluronan synthase 1; *IL-13*: Interleukin 13; *MMP3*: Matrix metallopeptidase 3; *SLURP1*: Secreted LY6/PLAUR domain containing 1

Status	Target	Drug (s)
**Upregulated in COVID-19-associated Tracheal Stenosis**	**MMP3**	• *MMP3 inhibitors* [33]: Actinonin [39], PD166793 [40], MMP Inhibitor V [41], MMP-3 Inhibitor VIII [42], MMP-3 Inhibitor V [43], MMP-2/3 Inhibitor II [44], UK 370,106 [45], UK 356,618 [46].
		• Retinoids [18]
	**CASP14**	• *Caspase inhibitors*: YVAD-FMK and ZVAD-FMK [47]
		• Retinoids [19]
	**HAS1**	• Pyrrolidine dithiocarbamate (NF-kappa-B inhibitor) [48]
		• *COX-2 inhibitors*: Rofecoxib and indomethacin [49]
		• Glucocorticoids [50]
		• Leflunomide [51]
		• Note: Retinoids may increase HAS2 and HAS3, but not HAS1 [52].
**Downregulated in COVID-19-associated Tracheal Stenosis**	**CAPN14**	• Recombinant IL-13 [53]
		• Recombinant CAPN14 [53]
	**SLURP1**	• Retinoids promote expression [20]
		• Note: IL-13 down regulates expression [54]
	**CRNN**	• Recombinant cornulin
		• Retinoids promote expression [21]
	**SPRR3**	• Hydroquinone [21]
		• Note: Retinoids decreases expression [55]
	**RPTN**	• Hydroquinone [21]
		• Note: Retinoids decreases expression [56]

Notably, retinol metabolism was also identified as the sole significantly downregulated cellular pathway (Supplementary File 1 - Table 2). Thus, we conducted a deep-dive into the genes directly involved in the metabolism and action of retinoic acid (active form of retinol). Gene expression appeared to indicate a state of compensation for relative retinoic acid deficiency, with CRABP1 (cellular retinoic acid-binding protein 1: functions to inhibit retinoic acid’s activity) being downregulated by a factor of 18.04 and CRABP2 (cellular retinoic acid-binding protein 1: functions to enable retinoic acid’s cellular activity) being upregulated by a factor of 99.96. In addition, RBP1 (retinol-binding protein 1: facilitates enzymatic conversion of retinol to retinoic acid) was upregulated by a factor of 22.02. The local retinoic acid deficiency may arise due to suppressed retinoic acid transport from the liver to the trachea: RBP4 (retinol-binding protein 4: functions to transport retinoic acid from the liver to peripheral tissues) was downregulated by a factor of 26.01. Moreover, enzymatic conversion of retinoid precursors to retinoic acid was also suppressed: RALDH2/ALDH1A2 (retinaldehyde dehydrogenase 2/ aldehyde dehydrogenase 1 family member A2: catalyzes synthesis of retinoic acid from retinaldehyde) was downregulated by a factor of 12.71.

In addition, we also compared the significantly dysregulated genes for Patient 1 vs. Patient 2. Genes that were uniquely upregulated in Patient 2 (vs. Patient 1) were involved in cellular pathways related to carcinogenesis.

The raw transcriptomic data is shown in Supplementary File 2.

## Discussion

**Fig. 1:**
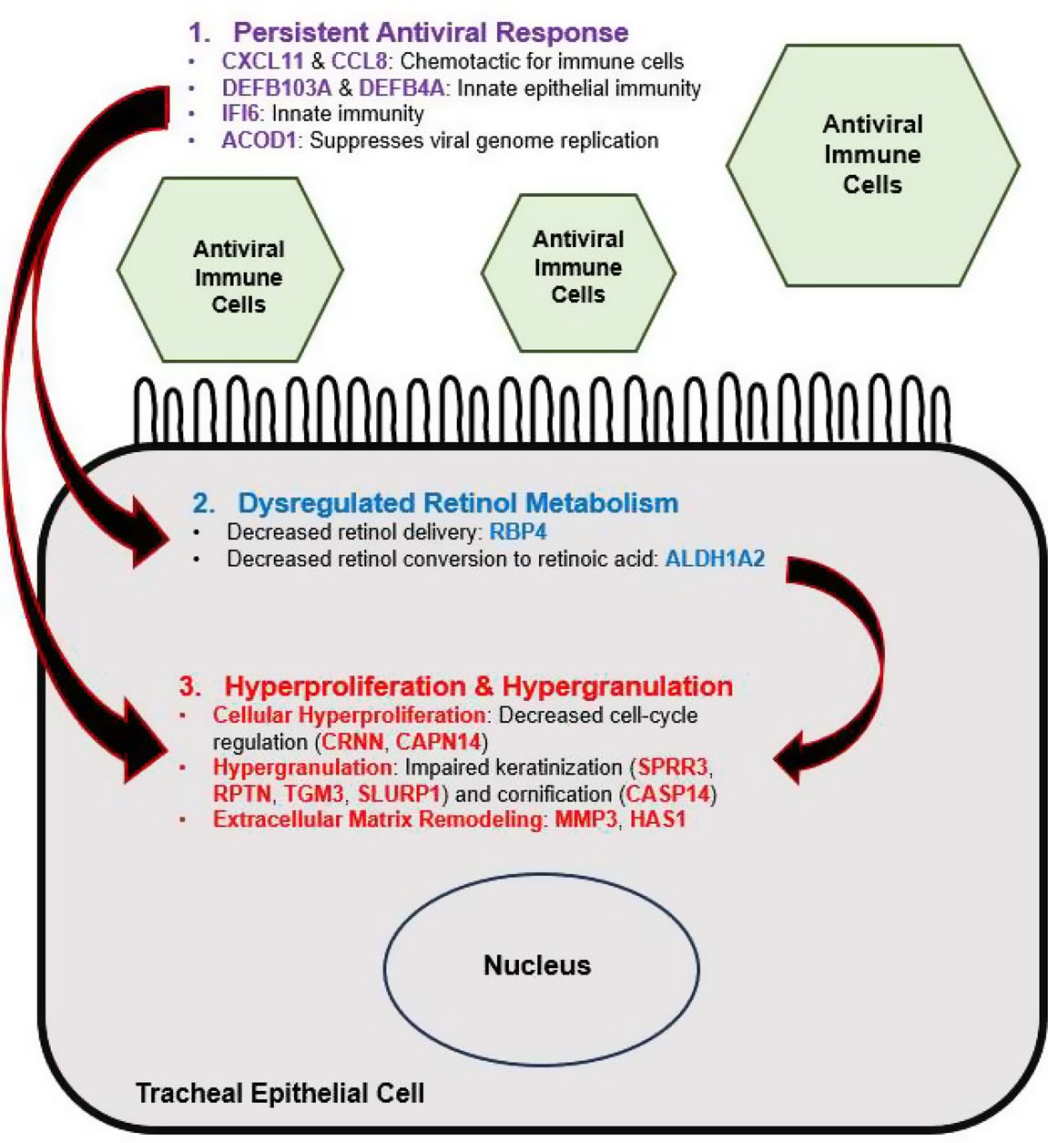
Persistent antiviral response may lead to dysregulated retinol metabolism; these collectively cause the hyperproliferation and hypergranulation characteristic of tracheal stenosis

This is the first study reporting patterns of gene expression in COATS. RNA sequencing analysis indicated an upregulation of genes and pathways involved in a persistent cellular antiviral response, confirming the notion that the pathophysiology of COATS features an infective component. Gene expression indicated cellular processes typical of tracheal stenosis, such as hyperproliferation (due to downregulated CRNN and CAPN14 causing decreased cell-cycle regulation), hypergranulation (due to downregulated SPRR3, RPTN, TGM3, and SLURP1 causing dysregulated keratinization), and extracellular matrix remodeling (upregulated MMPs and HAS1). Several of the upregulated genes indicated a cellular response to viral infection (CXCL11, CCL8, DEFB103A, IFI6, ACOD1, and DEFB4A). CXCL11 and CCL8 are involved in chemotaxis of immune cells, while the defensins (DEFB103A and DEFB4A) are broadly involved in innate immunity at the epithelial surface of the trachea. IFI6 plays an important role in the innate immunity against viruses, while ACOD1 also acts in an anti-viral capacity by suppressing replication of viral genomes. Our results also indicated downregulation of cellular pathways involved in retinol metabolism and suggested a state of relative retinoic acid deficiency. Figure 1 summarizes and synthesizes the proposed interactions between the key cellular processes contributing to COATS.

We performed a literature search to identify potential therapeutic options that could inhibit pathologically upregulated gene products or promote pathologically downregulated gene products (Table 3). Retinoic acid suppresses MMP3 [18] and CASP14 [19] (upregulated in COATS) and promotes SLURP1 [20] and CRNN [21] (downregulated in COATS). MMP3 and CASP14 may also serve as molecular targets for the prevention and management of COATS, as their expression may be suppressed using their respective inhibitors. Thus, retinoic acid, MMP3 inhibitors, and CASP14 inhibitors may hold therapeutic promise and should be prioritized for future investigation.

Retinoic acid is well-known for its role in maintaining epithelial integrity and promoting mucosal immunity [22]. Topical retinoic acid can improve the regeneration of a mucociliary respiratory epithelium after iatrogenic mucosal injury [23]. Retinoic acid has been shown to suppress MMPS [18] and CASP14 [19], while promoting expression of SLURP1 [20] and CRNN [21]. Moreover, retinoic acid has also been shown to promote cell-cell adhesion [24], which was also downregulated in the COATS tissue samples. Lastly, we also observed downregulation of pathways concerned with retinol metabolism. In fact, evidence suggests that retinoic acid depletion may be a common feature of COVID-19 infection [25, 26] and that administration of retinoic acid may reduce overall disease severity by targeting a variety of key molecular players [27–29]. Systemic retinoic acid can prevent virus-induced airway hyper-reactivity due to its anti-inflammatory and anti-viral effects [30]. This evidence collectively points towards retinoic acid being a potential adjunct therapy to prevent or treat COATS, and future studies must evaluate its benefits in animal models and clinical settings.

Other potential therapies that may be explored include MMP3 inhibitors and caspase inhibitors. MMP3, like other matrix metalloproteinases, plays a key role in extracellular matrix degradation during tissue remodeling that occurs as part of hyperproliferation and stenosis [31]. In COVID-19 infection, the upregulation of MMP3 has been identified previously as both a biomarker and a potential therapeutic target to prevent systemic complications of the disease [32, 33]. The main role of CASP14 (caspase-14) in the human body is the maintenance of the stratum corneum by promoting epidermal cornification [34]. However, formation of a cornified layer has been shown to occur in tracheal stenosis [35], presumably leading to a severe and recalcitrant manifestation of the disease. Caspases are widely upregulated in severe and chronic forms of COVID-19 infection, and caspase inhibitors have been identified as possible therapeutic options for these conditions [36]. While CASP-14 specific inhibitors have not been explored, pan-caspase inhibitors may be used in COVID-19 [36]. Thus, MMP3 and caspase inhibition also warrant attention with regards to their role in preventing and managing COATS.

In conclusion, we identified three upregulated genes (MMP3, CASP14, and HAS1) and five downregulated genes (SPRR3, CRNN, CAPN14, SLURP1, and RPTN) that likely promote tracheal hypergranulation and hyperproliferation leading to COVID-19-associated tracheal stenosis (COATS). In addition, retinol metabolism pathways were dysregulated, and gene expression indicated a relative local retinoic acid deficiency. Given the presence of existing literature highlighting retinoic acid’s ability to favorably regulate these genes, improve cell-cell adhesion, and decrease overall disease severity in COVID-19, future studies must evaluate its utility for adjunctive management of COATS in animal models and clinical settings.

## Limitations

This study has limitations. First this research note presents data from only five samples from two patients. Second, genes upregulated in Patient 2 indicated possible carcinogenesis (may be indicative of early neoplastic processes attributable to the patient’s smoking history), which may confound results. Third, our work only discusses on the biological plausibility of potential therapeutic options, and future work is required to evaluate actual benefits of these drugs. Lastly, we did not evaluate changing trends in the gene expression at different timepoints within the continuum of the disease course.

### Electronic supplementary material

Below is the link to the electronic supplementary material.

Supplementary Material 1

Supplementary Material 2

## Data Availability

The data that support the findings of this study are available from the corresponding author upon reasonable request. The genomic data for the patients with COATS can be found in Supplementary File 2.
