# Supplementary material for: Gene expression profiles in COVID-19-associated tracheal stenosis indicate persistent anti-viral response and dysregulated retinol metabolism
Source: BMC Res Notes. 2024 May 16;17:140. doi: 10.1186/s13104-024-06775-y (PMC11100031; doi:10.1186/s13104-024-06775-y)
Supplement: Supplementary file 2 — Supplementary Material 2 [file 13104_2024_6775_MOESM2_ESM.docx]

**Gene Expression Profiles in COVID-19-associated Tracheal Stenosis indicate** **Persistent Anti-Viral Response and Dysregulated Retinol Metabolism**

**Supplementary File 1**

| **Supplementary Table 1: Patient characteristics** | | | | | | |
| --- | --- | --- | --- | --- | --- | --- |
| **Subject** | **Age at Index Visit** | **Sex** | **Comorbids** | **Tracheal Stenosis Etiology** | **Interventions prior to Index Visit** | **Number of Samples Obtained** |
| **Patient 1** | 37 years | Female | ● Obstructive sleep apnea  ● Hypertension | Tracheostomy during COVID-19 infection | Spray cryotherapy and balloon dilation x 3 | 2 |
| **Patient 2** | 36 years | Female | ● Interstitial lung disease  ● Smoking | Prolonged intubation during COVID-19 infection. | None | 3 |

| **Supplementary Table 2: Notable changes in KEGG cellular pathways in COVID-19-associated tracheal stenosis** | | | |
| --- | --- | --- | --- |
| **Status of Pathway** | **Specific Pathway** | **KEGG Identification Number** | **Bonferroni P-Values** |
| **Upregulated** | Coronavirus disease 2019 infection | KEGG: 05171 | 1.8 $\times$ 10^-2^ |
|  | Non-specific viral infection  ● Influenza A virus  ● Human papillomavirus  ● Hepatitis C virus  ● Epstein-Barr virus  ● Measles virus | ● KEGG: 01564  ● KEGG: 05165  ● KEGG: 05160  ● KEGG: 05165  ● KEGG: 05162 | ● 1.2 $\times$ 10^-3^  ● 1.2 $\times$ 10^-3^  ● 1.5 $\times$ 10^-3^  ● 2.4 $\times$ 10^-2^  ● 1.2 $\times$ 10^-4^ |
|  | Cytokine-cytokine receptor interaction | KEGG: 04060 | 3.2 $\times$ 10^-2^ |
|  | Viral protein interaction with cytokine and cytokine receptor | KEGG: 04061 | 3.2 $\times$ 10^-2^ |
|  | NOD (nucleotide oligomerization domain)-like receptor signaling pathways | KEGG: 04621 | 1.8 $\times$ 10^-2^ |
| **Downregulated** | Retinol metabolism | KEGG: 00830 | 1.2 $\times$ 10^-4^ |

| **Supplementary Table 3: Notable changes in UniProt Key Words biological processes in COVID-19-associated tracheal stenosis** | | | |
| --- | --- | --- | --- |
| **Status of Process** | **Specific Process** | **Identification Number** | **Bonferroni P-Values** |
| **Upregulated** | Antiviral Response  ● Antiviral defense  ● Immunity  ● Innate immunity | ● GO: 0051607  ● GO: 0002376  ● GO: 0045087 | ● 7.3 $\times$ 10^-18^  ● 8.4 $\times$ 10^-12^  ● 1.3 $\times$ 10^-14^ |
|  | Inflammatory response | GO: 0006954 | 2.7 $\times$ 10^-2^ |
|  | Chemotaxis | GO: 0006935 | 2.7 $\times$ 10^-2^ |
|  | Angiogenesis | GO: 0001525 | 2.7 $\times$ 10^-2^ |
|  | Collagen degradation | GO: 0030574 | 8.7 $\times$ 10^-3^ |
| **Downregulated** | Cell adhesion | GO: 0007155 | 1.1 $\times$ 10^-4^ |
|  | Ion transport | GO: 0006811 | 1.8 $\times$ 10^-2^ |
